# Symptomatic Course of Foot Osteoarthritis Phenotypes: An 18‐Month Prospective Analysis of Community‐Dwelling Older Adults

**DOI:** 10.1002/acr.23502

**Published:** 2018-05-23

**Authors:** Thomas J. Downes, Linda Chesterton, Rebecca Whittle, Edward Roddy, Hylton B. Menz, Michelle Marshall, Martin J. Thomas

**Affiliations:** ^1^ Arthritis Research UK Primary Care Centre, Research Institute for Primary Care & Health Sciences Keele University Keele Staffordshire UK; ^2^ Haywood Academic Rheumatology Centre Staffordshire and Stoke‐on‐Trent Partnership NHS Trust Haywood Hospital Burslem Staffordshire UK; ^3^ La Trobe Sport and Exercise Research Centre School of Allied Health La Trobe University Bundoora Victoria Australia

## Abstract

**Objective:**

Osteoarthritis (OA) is a heterogeneous disease, and symptom progression at the foot is unclear. This study investigated the symptomatic course of 3 predefined foot OA phenotypes over an 18‐month period.

**Methods:**

The Clinical Assessment Study of the Foot is a community‐based cohort of adults ages ≥50 years in North Staffordshire, UK. Participants who reported foot pain in a postal health survey and underwent radiographic assessment were mailed an 18‐month followup survey. Changes in descriptive and symptomatic outcomes over 18 months were compared across the 3 phenotypes to determine within‐phenotype changes and between‐phenotype differences.

**Results:**

Of 533 participants at baseline, 478 (89.7%) responded at 18 months. All 3 phenotypes showed small within‐phenotype improvements in mean foot pain severity (scale range 0–10, where 0 = no pain and 10 = worst pain): no or minimal foot OA (18 months 4.0, mean change −1.15 [95% confidence interval (95% CI) −1.46, −0.83]), isolated first metatarsophalangeal (MTP) joint OA (18 months 4.1, mean change −0.60 [95% CI −1.11, −0.10]), and polyarticular foot OA (18 months 5.1, mean change −0.77 [95% CI −1.42, −0.12]). The isolated first MTP joint OA phenotype had an increased likelihood of hallux valgus in the left foot (adjusted odds ratio 2.96 [95% CI 1.23, 7.12]) compared to the no or minimal foot OA phenotype.

**Conclusion:**

Three foot OA phenotypes showed few descriptive or symptomatic changes over 18 months. Future clinical trials should consider that people recruited with mild‐to‐moderate symptomatic foot OA appear likely to remain relatively stable with usual care. Longer‐term followup using additional time points is required to describe further the natural history of foot OA.

## Introduction

Osteoarthritis (OA) is a multifactorial synovial joint disease, characterized by emerging clinical and structural subphenotypes, which, once they are fully explained, may facilitate more targeted treatment approaches 1. Most recently, epidemiologic observations of OA have extended to the foot, with symptomatic radiographic foot OA estimated to affect 1 in 6 adults ages 50 years and older 2. Despite recent evidence supporting the contribution of OA to foot pain, distinct progressive and nonprogressive symptomatic courses observed at the knee 3, hip 4, and hand 5 have yet to be investigated at the foot. Although only 1 prospective study has examined the progression of radiographic foot OA 6, the progression of symptoms among individuals with symptomatic radiographic foot OA remains unclear.

**Significance & Innovations**

- This is the first investigation of symptomatic change over time in patients with radiographically defined foot osteoarthritis (OA).
- Despite varying degrees of radiographic severity across phenotypes, few symptomatic changes over 18 months were observed within or between phenotypes.
- Future clinical trials should consider that people recruited with mild‐to‐moderate symptomatic foot OA appear likely to remain relatively stable with usual care over 18 months.

Using latent class analysis, we have recently identified 3 distinct foot OA phenotypes based on the radiographic scoring of 5 foot joints (first metatarsophalangeal [MTP] joint, first and second cuneometatarsal joint, navicular first cuneiform joint, and talonavicular joint) 7. These include an isolated first MTP joint OA phenotype and a polyarticular foot OA phenotype; both are found to be distinct from a phenotype with no or minimal foot OA 7. Cross‐sectionally, the polyarticular foot OA phenotype demonstrated more pain and functional limitation than the other 2 phenotypes, as well as stronger associations with female sex, higher body mass index (BMI), and nodal hand OA 7. The present analyses extend our investigations of these distinctive foot OA phenotypes to describe their natural history over time. Specifically, the aim of this study was to investigate the symptomatic course of these predefined foot OA phenotypes over an 18‐month period. Eighteen months is sufficient to detect a clinically meaningful change in OA if such a change is present 1. We hypothesized that symptoms would be relatively stable over 18 months, but that the polyarticular foot OA phenotype would demonstrate a trend toward worsening symptoms.

## Materials and methods

### Design and study population

Data from the Clinical Assessment Study of the Foot (CASF) were used. The CASF is a community‐based cohort of adults ages 50 years and older, registered with 1 of 4 general practices in North Staffordshire, UK. A full protocol has been reported previously 8. Briefly, participants who reported foot pain in the previous 12 months in a baseline postal health survey were invited to attend a research clinic, where weight‐bearing anteroposterior and lateral radiographs of both feet were obtained. Participants with no foot radiographs or an inflammatory arthropathy (nonspecific inflammatory arthritis, rheumatoid arthritis, or psoriatic arthritis), as identified from medical records or clinical radiology reports, were excluded from the analyses. A followup survey was mailed to participants 18 months after their visit. Participants who did not respond to the 18‐month followup survey after 2 weeks were sent a reminder postcard. Participants who did not respond after 4 weeks from the initial mailing were sent a repeat survey. Nonresponders to the repeat survey were further invited to complete a shortened minimal data collection (MDC) questionnaire designed to capture key outcome data. MDC was completed by telephone, or if that option was unavailable, by mail 8. Ethical approval was obtained from the Coventry Research Ethics Committee (REC reference number 10/H1210/5), and all participants provided written informed consent. For this analysis, we retained participants in their previously assigned baseline foot OA phenotypes based on their radiographic characteristics: no or minimal foot OA, isolated first MTP joint OA, and polyarticular foot OA 7.

### Descriptive and symptomatic outcomes

Data collected from baseline only included age, sex, and BMI (calculated from height and weight measured at the baseline research clinic visit) 8. Data collected from both the baseline health survey and the 18‐month followup survey included the following: foot pain severity in the previous month, using a 0–10 numeric rating scale (NRS; 0 = no pain and 10 = worst possible pain); Rasch‐transformed Manchester Foot Pain and Disability Index (MFPDI), which derived an interval‐level scale from the original 3‐part ordinal MFPDI responses 9, 10; Short Form 12 (SF‐12) physical component summary (PCS) and mental component summary (MCS) scores 11; Hospital Anxiety and Depression Scale (HADS) 12; frequent foot pain in the previous month; dissatisfaction with the persistence of foot symptoms; presence of hip and/or knee pain in the previous year; and hallux valgus. Frequent foot pain was defined as participants reporting pain or aching or stiffness in their feet on most days or all days in the previous month. Dissatisfaction with the persistence of foot symptoms was defined as participants being very dissatisfied or somewhat dissatisfied with spending the rest of their lives with their current foot symptoms. Hallux valgus was categorized as unilateral or bilateral using a validated self‐report line‐drawing instrument 13. Participants chose 1 of 5 line drawings that best depicted the appearance of each foot. Each line drawing sequentially increased the hallux valgus angle by 15 degrees, with the 3 more severe illustrations categorized as hallux valgus 13.

Additional data collected at 18‐month followup only included the following: perceived global change in foot pain over 18 months since baseline clinic attendance, which was categorized as improved, unchanged, and worsened; foot injury and foot operation in the previous 18 months; and use of services or treatments because of foot pain in the previous 18 months. Services or treatments included at least 1 of the following: physical therapy, hospital specialist, podiatrist, chiropodist, acupuncture, osteopath or chiropractor, prescription of drugs, foot operation, foot injection, or general practitioner (family doctor).

Symptomatic outcomes contained in both the full survey and in the MDC included perceived global change in foot pain, MFPDI pain and function scores, and frequent foot pain in the previous month.

### Statistical analysis

Descriptive and symptomatic outcomes were analyzed with baseline and 18‐month data to investigate within‐phenotype changes and between‐phenotype differences. Statistical significance was determined as *P* < 0.05. Changes over time within phenotypes were examined using McNemar's test for dichotomous variables, and paired *t*‐testing was used for continuous variables. Between‐phenotype differences were examined using binary logistic regression for dichotomous outcomes and linear regression for continuous outcomes. The no or minimal foot OA phenotype was used as the reference category for the regression analyses. Estimates were adjusted for baseline scores, and for the potential confounders age, sex, and BMI, due to observed between‐phenotype differences at baseline 7. Using data at 18 months only, differences between the 3 phenotypes at 18 months were examined using chi‐square testing. All analyses were conducted using SPSS, version 21.

## Results

### Study population

Of the 533 participants at baseline, after exclusions for no foot radiographs (n = 3) and inflammatory arthritis (n = 24), 478 returned the 18‐month followup survey (89.7% response rate), of which 307 (64.2%) had no or minimal foot OA, 101 (21.1%) isolated first MTP joint OA, and 70 (14.6%) polyarticular foot OA. Participants who returned the followup survey were generally similar to those lost to followup. However, those lost to followup were more likely to have dissatisfaction with the persistence of foot symptoms, hip pain, and knee pain (see Supplementary Table 1, available on the *Arthritis Care & Research* web site at http://onlinelibrary.wiley.com/doi/10.1002/acr.23502/abstract). Reasons for loss to followup are outlined in Supplementary Figure 1 (available at http://onlinelibrary.wiley.com/doi/10.1002/acr.23502/abstract).

### Within‐phenotype changes

Overall, all 3 phenotypes showed small mean and percentage changes between baseline and 18 months (Table 1). Small but statistically significant improvements were observed for mean foot pain severity within all 3 phenotypes: no or minimal foot OA (18 months 4.0, mean change −1.15 [95% confidence interval (95% CI) −1.46, −0.83]), isolated first MTP joint OA (18 months 4.1, mean change −0.60 [95% CI −1.11, −0.10]), and polyarticular foot OA (18 months 5.1, mean change −0.77 [95% CI −1.42, −0.12]). Additionally, individuals with no or minimal foot OA showed a statistically significant improvement in mean Rasch‐transformed MFPDI pain score (18 months −0.6, mean change −0.29 [95% CI −0.46, −0.12]), mean SF‐12 PCS score (18 months 40.9, mean change +1.71 [95% CI 0.66, 2.75]), mean HADS anxiety score (18 months 6.5, mean change −0.58 [95% CI −0.96, −0.20]), and the proportion of participants reporting frequent foot pain in the previous month (18 months 39.1%, change −10.7%). However, a greater proportion of participants in the no or minimal foot OA phenotype group reported hip pain at 18 months than at baseline (18 months 58.8%, change +5.9%). Individuals in the isolated first MTP joint OA phenotype group reported dissatisfaction with foot symptoms persisting less frequently (18 months 30.5%, change −12.7%). The polyarticular foot OA phenotype showed statistically significant improvement in mean HADS anxiety scores (18 months 6.5, mean change −0.72 [95% CI −1.37, −0.08]).

**Table 1:** Changes in selected outcomes from baseline to 18 months for the 3 foot OA phenotypes^a^

	No or minimal foot OA		Isolated first MTP joint OA		Polyarticular foot OA	
	18‐month score, mean ± SD or no. (%)	Mean change (95% CI) or % change (*P*)	18‐month score, mean ± SD or no. (%)	Mean change (95% CI) or % change (*P*)	18‐month score, mean ± SD or no. (%)	Mean change (95% CI) or % change (*P*)
Foot pain severity in previous month (0–10 NRS)^b^	4.0 ± 2.8	−1.15 (−1.46, −0.83)^c^	4.1 ± 2.8	−0.60 (−1.11, −0.10)^c^	5.1 ± 2.5	−0.77 (−1.42, −0.12)^c^
Rasch‐transformed MFPDI pain score^d^	−0.6 ± 1.7	−0.29 (−0.46, −0.12)^c^	−0.8 ± 1.6	−0.22 (−0.51, 0.07)	0.1 ± 1.4	−0.12 (−0.40, 0.16)
Rasch‐transformed MFPDI function score^d^	−0.9 ± 2.1	−0.03 (−0.20, 0.14)	−1.0 ± 2.1	+0.01 (−0.30, 0.31)	0.2 ± 2.1	+0.07 (−0.27, 0.41)
SF‐12 PCS score^d^	40.9 ± 12.2	+1.71 (0.66, 2.75)^c^	40.8 ± 11.1	−0.34 (−2.29, 1.61)	37.6 ± 11.8	+1.12 (−0.98, 3.22)
SF‐12 MCS score^d^	49.5 ± 10.7	+0.03 (−1.13, 1.19)	50.2 ± 10.6	−1.04 (−2.98, 0.90)	47.9 ± 11.9	−0.96 (−3.72, 1.79)
HADS anxiety score^d^	6.5 ± 4.3	−0.58 (−0.96, −0.20)^c^	5.9 ± 4.2	−0.55 (−1.23, 0.13)	6.5 ± 3.8	−0.72 (−1.37, −0.08)^c^
HADS depression score^d^	5.1 ± 3.8	−0.26 (−0.55, 0.35)	4.6 ± 3.6	−0.13 (−0.67, 0.41)	6.1 ± 3.8	−0.15 (−0.72, 0.41)
Frequent foot pain in the previous month^e^	116 (39.1)	−10.7 (< 0.01)^c^	38 (38.0)	−10.0 (0.1)	38 (55.9)	−13.2 (0.12)
Dissatisfaction with foot symptoms persisting^f^	122 (40.9)	−6.1 (0.08)	29 (30.5)	−12.7 (0.04)^c^	32 (48.5)	−13.6 (0.12)
Bilateral hallux valgus^g^	49 (17.0)	+0.7 (0.87)	27 (27.8)	0.0 (1.00)	22 (32.8)	−6.0 (0.45)
Unilateral hallux valgus, left foot^g^	21 (7.3)	−2.4 (0.27)	12 (12.4)	+6.2 (0.07)	8 (11.9)	+1.5 (1.00)
Unilateral hallux valgus, right foot^g^	39 (13.5)	+0.7 (0.87)	9 (9.3)	−1.0 (1.00)	7 (10.4)	+2.9 (0.75)
Hip pain in the previous year	171 (58.8)	+5.9 (0.04)^c^	60 (61.2)	+7.1 (0.19)	45 (68.2)	0.0 (1.00)
Knee pain in the previous year	218 (74.7)	+2.4 (0.38)	77 (78.6)	+5.1 (0.41)	55 (83.3)	−6.1 (0.29)

a OA = osteoarthritis; MTP = metatarsophalangeal; 95% CI = 95% confidence interval; NRS = numeric rating scale; MFPDI = Manchester Foot Pain and Disability Index; SF‐12 = Short Form 12; PCS = physical component summary; MCS = mental component summary; HADS = Hospital Anxiety and Depression Scale.

b 0 = no pain and 10 = worst possible pain.

c Statistically significant (*P* < 0.05).

d Higher MFPDI scores indicate more pain/functioning; higher SF‐12 PCS and MCS scores indicate better health; and higher HADS scores indicate worse psychiatric ratings.

e Defined as frequent pain, aching, or stiffness on all or most days in the previous month.

f Defined as participants being very or somewhat dissatisfied with the foot symptoms persisting for the rest of their lives.

g Hallux valgus was defined according to Roddy et al's 13 self‐report instrument and dichotomized definition.

### Between‐phenotype differences

Following adjustment for baseline scores, age, sex, and BMI, generally small between‐phenotype differences were seen over 18 months between the isolated first MTP joint OA and polyarticular foot OA phenotypes in relation to the reference category of the no or minimal foot OA phenotype (Table 2). The isolated first MTP joint OA phenotype was significantly more likely than the no or minimal foot OA phenotype to report unilateral hallux valgus in the left foot at 18 months (adjusted odds ratio 2.96 [95% CI 1.23, 7.12]).

**Table 2:** Between‐phenotype differences for the isolated first MTP joint OA and polyarticular foot OA phenotype at 18 months using the no or minimal foot OA phenotype as the reference category^a^

	Isolated first MTP joint OA			Polyarticular foot OA		
	β_adj_ b	OR_adj_ b	95% CI	β_adj_ b	OR_adj_ b	95% CI
Foot pain severity in previous month (NRS)^c^	0.29		−0.27, 0.85	0.46		−0.21, 1.13
Rasch‐transformed MFPDI pain score^d^	0.02		−0.28, 0.33	0.37		−0.01, 0.74
Rasch‐transformed MFPDI function score^d^	0.00		−0.31, 0.31	0.27		−0.11, 0.65
SF‐12 PCS score^d^	−1.04		−2.96, 0.88	0.69		−3.11, 1.73
SF‐12 MCS score^d^	−0.49		−2.57, 1.59	−0.69		−3.32, 1.94
HADS anxiety score^d^	−0.08		−0.76, 0.60	−0.16		−0.96, 0.65
HADS depression score^d^	−0.06		−0.60, 0.49	0.20		−0.46, 0.86
Frequent foot pain in the previous month^e^		0.94	0.57, 1.56		1.43	0.79, 2.59
Dissatisfaction with foot symptoms^f^		0.64	0.38, 1.09		1.00	0.55, 1.82
Bilateral hallux valgus^g^		1.45	0.75, 2.81		1.26	0.58, 2.72
Unilateral hallux valgus, left foot^g^		2.96^h^	1.23, 7.12^h^		2.18	0.76, 6.30
Unilateral hallux valgus, right foot^g^		0.67	0.30, 1.52		0.77	0.31, 1.95
Hip pain in the previous year		0.84	0.48, 1.49		0.94	0.46, 1.93
Knee pain in the previous year		0.82	0.43, 1.60		1.55	0.67, 3.59

a MTP = metatarsophalangeal; OA = osteoarthritis; adj = adjusted; OR = odds ratio; 95% CI = 95% confidence interval; NRS = numeric rating scale; MFPDI = Manchester Foot Pain and Disability Index; SF‐12 = Short Form 12; PCS = physical component summary; MCS = mental component summary; HADS = Hospital Anxiety and Depression Scale.

b Adjusted for baseline scores, age, sex, and body mass index.

c 0 = no pain and 10 = worst possible pain.

d Higher MFPDI indicate more pain/functioning; higher SF‐12 PCS and MCS scores indicate better health; and higher HADS scores indicate worse psychiatric ratings.

e Defined as frequent pain, aching, or stiffness on all or most days in the previous month.

f Defined as participants being very or somewhat dissatisfied with the foot symptoms persisting for the rest of their lives.

g Hallux valgus was defined according to Roddy et al's 13 self‐report instrument and dichotomized definition.

h Statistically significant (*P* < 0.05).

There were no statistically significant differences in perceived global change in foot pain or foot injuries incurred over 18 months among the foot OA phenotypes (Table 3). However, a higher proportion (40.6%) of individuals in the polyarticular foot OA phenotype perceived that their foot pain had worsened compared to the first MTP joint OA (27.0%) and no or minimal foot OA (27.9%) phenotypes. Approximately half of the participants in each phenotype reported using a service or treatment for foot pain in the preceding 18 months. The proportion of participants reporting a foot operation during this period was very low (≤4.0%) for each phenotype.

**Table 3:** Descriptive and symptomatic outcomes analyzed using only 18‐month data^a^

Outcome	No or minimal foot OA	Isolated first MTP joint OA	Polyarticular foot OA	*P*
Perceived global change in foot pain in previous 18 months				0.108
Improved	95 (31.6)	26 (26.0)	13 (18.8)	
Unchanged	122 (40.5)	47 (47.0)	28 (40.6)	
Worsened	84 (27.9)	27 (27.0)	28 (40.6)	
Foot injury in previous 18 months	18 (6.2)	8 (8.1)	7 (10.8)	0.404
Use of services or treatment for foot pain in previous 18 months^b^	146 (48.5)	45 (45.5)	37 (54.4)	0.519
Foot operation in previous 18 months	1 (0.3)	4 (4.0)	2 (2.9)	c

a Values are the number (%) unless otherwise indicated. OA = osteoarthritis; MTP = metatarsophalangeal.

b Services or treatment for foot pain included at least 1 of the following: physical therapy, hospital specialist, podiatrist, chiropodist, acupuncture, osteopath or chiropractor, prescription drugs, foot operation, foot injection, or general practitioner (family doctor).

c Not calculated, as expected cell counts were below 5 for all 3 phenotypes.

## Discussion

This study investigated the symptomatic course of 3 foot OA phenotypes over an 18‐month period. The main finding from this study was a general trend for slight improvements of health outcomes across all 3 foot OA phenotypes, with small but statistically significant reductions in foot pain severity in particular. Few between‐phenotype differences occurred over the 18‐month period.

In absolute terms, the reduction in pain severity across the 3 phenotypes was small (range 0.60–1.15 NRS points), with all observed values under the accepted 2‐point reduction threshold applied to denote a clinically important difference in musculoskeletal pain 14. Therefore, while observed changes in pain severity were statistically significant, they are unlikely to represent a clinically meaningful change for the participants. Furthermore, it is impossible to know with certainty whether and how improvements in foot pain severity correspond to sites of radiographic OA. Potential explanations for the observed reduction in pain may include increased awareness and prioritization of foot pain after enrollment into the CASF study and regression to the mean. However, the polyarticular foot OA group had a higher proportion of participants that indicated worsening in their global foot pain over 18 months compared to the other phenotypes, albeit not significantly.

A trend of pain improvement at the first followup measurement is consistent with improvements in knee pain trajectories observed in adults with knee OA 3. Collins et al found that, following initial improvement from baseline, all knee pain trajectories remained relatively stable over the remaining 5‐year followup 3. With only 1 followup time point in this study, it is uncertain whether the small changes in foot pain observed over 18 months are representative of the long‐term clinical course of foot OA. Furthermore, pain trajectories are not always stable and may fluctuate over time, as previously observed for hip OA 4. Our findings suggest mild‐to‐moderate symptomatic foot OA progression is unlikely to be rapid over 18 months, and management can be monitored in primary care without the need for routine referral to secondary care. Future research directed at identifying individuals most likely to have unfavorable prognosis, who would benefit from timely onward referral, would appear to be important.

Between‐phenotype comparisons identified little difference between the foot OA phenotypes in relation to their descriptive and symptomatic characteristics. Following adjustment for potential confounders, there was only 1 statistically significant between‐phenotype difference: an increased likelihood of unilateral hallux valgus in the left foot for the isolated first MTP joint OA phenotype compared to the no or minimal foot OA phenotype. Comparison of actual numbers revealed that overall there were 6 new cases of unilateral hallux valgus in the left foot for the isolated first MTP joint OA phenotype, and 7 fewer cases for the no or minimal foot OA phenotype. While the identification of new cases over an 18‐month period is a possibility, the progressive nature of hallux valgus makes an observed reduction in severity appear implausible. The number of reported foot operations and new bilateral hallux valgus cases, suggesting progression from unilateral to bilateral hallux valgus, at 18 months were insufficient to account for this observation. Misclassification of self‐reported hallux valgus may therefore account for some of the reported changes over 18 months, particularly when participants reported borderline hallux valgus. Despite the hallux valgus line‐drawing instrument previously demonstrating good reliability over a 6‐month period 13, we did not assess reliability again at 18 months, and it is plausible that this was lower than that previously reported. Indeed, the wide 95% CI for the odds ratio of the unilateral hallux valgus in the left foot reflects an imprecise estimate. Therefore, although these findings may indicate that the first MTP joint OA phenotype is a risk factor for the development of unilateral hallux valgus in the left foot, the finding is possibly spurious and should be interpreted with caution.

The data from this study were derived from CASF, which has a source population broadly representative of the British population, despite having a lower proportion of ethnic minorities 8. By identifying participants from CASF with foot pain over the previous year, this study provides a sample broadly representative of the British population with foot pain. Additionally, there was a high retention of participants at 18 months (89.7%). However, some limitations need to be considered. First, participants were likely to have foot pain across multiple foot areas. Foot pain can lead to compensatory changes in gait and foot function, thus increasing the risk of pain in other areas of the foot 15. Therefore, whether changes in reported foot pain severity related to the same pain sites from baseline to followup is uncertain. Second, participants lost to followup had a trend for being more dissatisfied with foot symptoms persisting, while also having more hip and knee pain (see Supplementary Table 1, available on the *Arthritis Care & Research* web site at http://onlinelibrary.wiley.com/doi/10.1002/acr.23502/abstract). This suggests that participants lost to followup had more widespread joint pain. Although this is unlikely to have influenced the relative differences between the phenotypes, it may have resulted in an underestimation of absolute symptom severity. Third, participants were allocated to foot OA phenotypes at baseline; therefore, whether participants transitioned between phenotypes over time is uncertain.

In conclusion, to our knowledge this is the first study to investigate symptomatic changes in patients with radiographic foot OA over time. Although our findings suggest a general statistical trend toward slight symptomatic improvement, this is unlikely to be clinically meaningful. Few between‐phenotype differences were observed, and a statistically significant finding of more prevalent unilateral hallux valgus in the isolated first MTP joint OA phenotype may be an artefact of misclassification. Future clinical trials should consider that people recruited with mild‐to‐moderate symptomatic foot OA appear likely to remain relatively stable with usual care. Additional followup over a longer time period is needed to understand further the natural history of foot OA and whether the course of foot symptoms differs between different phenotypes.

## Author contributions

All authors were involved in drafting the article or revising it critically for important intellectual content, and all authors approved the final version to be submitted for publication. Dr. Thomas had full access to all of the data in the study and takes responsibility for the integrity of the data and the accuracy of the data analysis.

### Study conception and design

Downes, Chesterton, Roddy, Menz, Marshall, Thomas.

### Acquisition of data

Roddy, Marshall, Thomas.

### Analysis and interpretation of data

Downes, Chesterton, Whittle, Roddy, Menz, Marshall, Thomas.

## Supporting information

Supplementary Table 1
Supplementary Figure 1
