# Supplementary material for: Symptomatic Course of Foot Osteoarthritis Phenotypes: An 18‐Month Prospective Analysis of Community‐Dwelling Older Adults
Source: Arthritis Care Res (Hoboken). 2018 May 23;70(7):1107–12. doi: 10.1002/acr.23502 (PMC6067068; doi:10.1002/acr.23502)
Supplement: Supplementary file 1 — Supplementary Table 1 Supplementary Figure 1 [file ACR-70-1107-s001.docx]

Supplementary material

**Supplementary Table 1: Comparison of baseline outcomes between responders to the 18-month follow-up health survey and responders to the Minimal Data Collection questionnaire with non-responders at 18-month follow-up.**

|  | **18-month cohort with full survey and MDC (n= 478)** | **Lost to follow-up from baseline analysis (n= 55)** |
| --- | --- | --- |
| Age: mean (SD) | 64.7 (8.1) | 67.0 (10.8) |
| Female: n (%) | 266 (55.6) | 32 (58.2) |
| BMI: mean (SD) | 30.3 (5.6) | 30.9 (6.1) |
| Rasch-transformed MFPDI pain: mean (SD) | -0.3 (1.5) | 0.0 (1.7) |
| Rasch-transformed MFPDI function: mean (SD) | -0.7 (2.1) | 0.1 (2.4) |
| Frequent foot pain in the previous month ^a^: n (%) | 249 (52.8) | 30 (55.6) |
|  | **18-month cohort with full survey (n= 468)** | **Lost to follow-up from baseline analysis and MDC (n= 65)** |
| Foot pain severity (0-10 Numeric rating scale) in the previous month ^b^: mean (SD) | 5.2 (2.6) | 5.9 (2.5) |
| SF-12 PCS: mean (SD) | 39.2 (12.1) | 32.2 (12.0) |
| SF-12 MCS: mean (SD) | 49.6 (10.6) | 45.0 (12.7) |
| HADS anxiety: mean (SD) | 7.0 (4.3) | 7.8 (4.8) |
| HADS depression: mean (SD) | 5.3 (3.8) | 7.0 (4.6) |
| Dissatisfaction with foot symptoms persisting ^c^: n (%) | 224 (48.4) | 36 (59.0) |
| Bilateral hallux valgus ^d^: n (%) | 104 (22.4) | 14 (21.5) |
| Unilateral hallux valgus ^d^ - left foot: n (%) | 41 (8.8) | 8 (12.3) |
| Unilateral hallux valgus ^d^ - right foot: n (%) | 54 (11.6) | 10 (15.4) |
| Hip pain in the previous year: n (%) | 257 (55.0) | 45 (69.2) |
| Knee pain in the previous year: n (%) | 350 (75.3) | 55 (84.6) |

*BMI= body mass index; HADS= Hospital Anxiety and Depression Scale (higher HADS score indicate worse psychiatric ratings); MCS= mental component summary; MDC= Minimal Data Collection questionnaire; MFPDI= Manchester Foot Pain and Disability Index (higher MFPDI scores indicate higher pain/ function); MTPJ= metatarsophalangeal joint; OA= osteoarthritis; PCS= physical component summary; SD= standard deviation; SF-12= 12-Item Short-Form Health Survey (higher SF-12 PCS and MCS scores indicated better health); ^a^= defined as frequent pain, aching or stiffness on all or most days in the previous month; ^b^= The numeric rating scale included verbal anchors of “no pain” at 0 and “pain as bad as could be” at 10; ^c^= defined as participants being very or somewhat dissatisfied with the foot symptoms persisting for the rest of their lives; ^d^= Hallux valgus was defined according to Roddy et al.’s [13] self-report instrument and dichotomised definition.*

Invited to baseline clinic (n= 1,634)

Attended baseline clinic (n= 560)

Responders to an 18-month Follow-up Health Survey (n= 478)

- *Minimal Data Collection questionnaire (n= 10)*
- *18-month follow-up health survey (n= 468)*

Excluded during mailing (n= 8)

- *Deaths and departures (n= 3)*
- *Ill health (n= 1)*
- *Return addressee unknown (n= 4)*

Refusals/ non-responders (n= 1,066)

- *Ill health (n= 27)*
- *Refused (n= 329)*
- *Non-responders (n= 710)*

Included in the baseline analysis (n= 533)

Excluded during radiographic assessment (n= 27)

- *Inflammatory arthropathy (n= 24)*
- *Missing radiograph data (n= 3)*

Lost to 18-month follow-up (n= 55)

- *No permission from new general practitioner (n= 6)*
- *Screened out by current general practitioner (n= 1)*
- *Died (n= 9)*
- *Terminally ill (n= 1)*
- *Health problems – refused (n= 1)*
- *Health problems – withdraw (n= 3)*
- *Refused (n= 6)*
- *Withdraw (n= 6)*
- *Non-response (n= 22)*

**Supplementary Figure 1: Flowchart illustrating study attrition from participants over 18 months in the CASF study (adapted reproduction from Roddy et al. (2015)).**
